# Impact of a Discontinuous Training Program on Sedentary Behavior in Italian Type 2 Diabetes Older Patients: The Results of the TRIPL-A Randomized Controlled Trial

**DOI:** 10.3390/healthcare12080848

**Published:** 2024-04-17

**Authors:** Anna Rita Bonfigli, Cristina Gagliardi, Olga Protic, Adrianapia Maria Lamedica, Maria Paola Luconi, Federica Turchi, Elena Tortato, Mirko Di Rosa, Francesco Lucertini, Liana Spazzafumo

**Affiliations:** 1 Scientific Direction, National Institute of Health and Science on Aging (IRCCS INRCA), 60127 Ancona, Italy; 2 Centre for Socio-Economic Research on Ageing, National Institute of Health and Science on Aging (IRCCS INRCA), 60124 Ancona, Italy; 3 Diabetology Unit, National Institute of Health and Science on Aging (IRCCS INRCA), 60127 Ancona, Italy; 4 Centre for Biostatistics and Applied Geriatric Clinical Epidemiology, National Institute of Health and Science on Aging (IRCCS INRCA), 60124 Ancona, Italy; m.dirosa@inrca.it; 5 Department of Biomolecular Sciences, University of Urbino Carlo Bo, 61029 Urbino, Italy; francesco.lucertini@uniurb.it

**Keywords:** type 2 diabetes, quality of life, physical activity, lifestyle, stages of change

## Abstract

**Background**: Physical activity is an important predictor of quality of life in older adults with type 2 diabetes (T2D). Unfortunately, most T2D adults adopt a sedentary lifestyle. The randomized, controlled TRIPL-A trial aims to verify the effect of a personalized, discontinuous exercise program on a sedentary lifestyle of T2D older adults. **Methods**: A total of 305 T2D patients (mean age ± SD: 68.8 ± 3.3 years) were divided into a control arm receiving only behavioral counseling and an intervention arm of an 18-month supervised discontinuous exercise program (ERS). The primary outcomes were the changes in sitting time (ST) and metabolic equivalent (MET) values, both evaluated by the International Physical Activity Questionnaire short form. A repeated measures ANOVA with Bonferroni correction for multiple comparisons was used to compare study outcomes. **Results**: The ST and MET differed significantly during the study compared to the control group (*p* = 0.028 and *p* = 0.004, respectively). In the intervention group, a decrease from baseline in ST at 6 months (*p* = 0.01) and an increase in MET values at 6 months (*p* = 0.01) up to 12 months (*p* < 0.01) were found. No significant differences were found for the other variables. **Conclusions**: Beneficial lifestyle changes were found within the first year of intervention. These results align with the theory of change.

## 1. Introduction

Regular physical activity (PA) is an important predictor of older people’s health and quality of life [1]. In contrast, physical inactivity is associated with an increased risk of numerous poor health conditions, including the most common ones, such as heart disease, diabetes, stroke, cancer, and sarcopenia [2]. The baby boomer generation, the largest cohort in history, is approaching retirement age [3]. The health status of this generation is a major public health challenge. Adopting and maintaining a regular exercise habit is challenging during aging [4]. For instance, people aged 70–79 years are about 50% less likely than those aged 50 to 59 years to be engaged in exercise and physical activity [5].

In this perspective, physicians can perform an important and valuable task, increasing the adherence rate to a physical activity program and helping patients manage the change in health-related behaviors through theoretical principles and techniques for behavior modification [4]. This model of change has been applied to dysfunctional behaviors, including those related to lifestyle-related disease. The stages of change represent both the phase of the process phase and the person’s behavioral characteristics [6]. Relapses consist of resuming problematic behavior one or more times, with a consequent return to a previous stage. This model emphasizes that change is a gradual, continuous, and dynamic process that responds to general rules but varies significantly from person to person. It also suggests that people are more ready for change before undertaking it.

Type 2 diabetes (T2D) is a growing global health problem [7]. Besides the question of the individual quality of life that this disease influences, the impact of T2D on the financial burden on healthcare systems is significant [8]. A recent meta-analysis demonstrated that exercise intervention improves inflammatory, metabolic, lipid markers, and insulin resistance in older adults with T2D [9]. The authors showed that the effects of different exercise interventions vary. Moreover, aerobic, high-intensity, and long-term exercise interventions significantly impact these biomarkers [10].

Structured PA is part of the T2D management plan [11]. PA prevents the onset of frailty and elicits numerous favorable effects that contribute to healthy aging [12,13]. Even with the well-known benefits of PA in improving blood glucose control and well-being, most older adults adopt a sedentary lifestyle [14,15].

Based on the model of change, during the first phases, many older adults with T2D may not be aware of the benefits of exercise or may feel daunted by the prospect of starting a new program. Educators or health professionals should provide information on the benefits of physical activity for T2D management, encourage awareness of the problem, and then set realistic goals to develop an action plan and suitable type of exercise.

Long-term diabetic education interventions often fail to yield significant improvements in blood glucose, even when proposed by qualified general practitioners (GPs) [16,17]. While available information primarily focuses on middle-aged patients, the literature’s data on older individuals with T2D remain scarce [18].

Exercise referral schemes (ERSs) have emerged as potential models for promoting PA among T2D patients [19,20]. In particular, applying ERSs could be a way to enhance PA levels, especially among sedentary older T2D subjects. In this approach, healthcare professionals refer patients to third-party services that tailor PA programs to individual needs. These services then prescribe and monitor the patient’s personalized program. However, the long-term effectiveness of ERSs in increasing physical activity remains limited [21]. To our knowledge, no randomized controlled trials have demonstrated the long-term effectiveness of ERS programs in educating sedentary older T2D patients to lead a more active lifestyle. Interestingly, several studies propose that integrating traditional methods with Web-Based Applications (WBAs) could enhance the effectiveness of interventions promoting PA in patients with T2D [22,23].

Considering the improvement in older people with T2D health status and their quality of life that could be improved with PA and, on the other hand, the impact on the cost on the health system that it could benefit, it would be worth fully finding an efficient way to stimulate a change in the sedentary lifestyle of older adults [19]. This work aims to verify if a randomized controlled trial with an ERS comprehending a discontinuous training program supported with WBAs could efficiently promote PA in primary care settings in sedentary T2D subjects.

## 2. Material and Methods

### 2.1. Study Design

The TRIPL-A is a randomized controlled trial with two parallel groups. The trial was retrospectively registered in the Australian New Zealand Clinical Trials Registry (ANZCTR) under the code ACTRN12618001164280.

In a clinical trial, 305 T2D older patients were randomly assigned to either the control or the intervention group in a 1:1 ratio. A trained physician performed the intervention on the subjects.

The randomization process used a computerized permuted block method. The statisticians from the INRCA Biostatistical Center created the randomization code. To ensure unbiased allocation, the trial staff did not know the randomization code or procedures. The actual group assignment occurred after all baseline tests had been completed. Due to the apparent differences between the interventions, neither staff nor participants were blinded to group allocation. However, before data analysis, patient allocations and identification numbers were replaced by categorical variables whose meaning remained unknown to data analysts.

**Figure 1:**
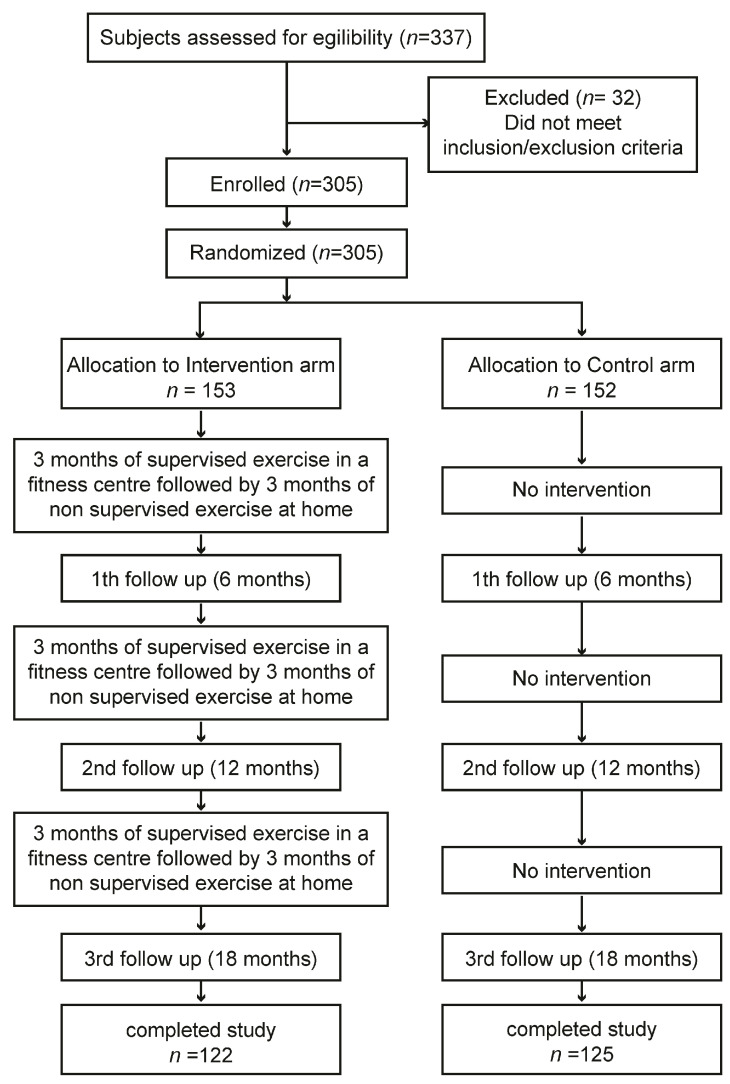
Schematic representation of study flow chart. From a total of 305 enrolled subjects, 247 patients completed the study. The overall dropout rate calculated at the end of the Trial was equal to 19%.

The TRIPL-A clinical study protocol obtained the favorable opinion of the Marche Regional Ethics Committee (ID: CERM 2015 333 IN 15011) on 22 October 2015. We described the study protocol previously in detail, comprehending the power analysis used to determine the sample size [24]. To detect a difference of 75 min per day in mean sitting times between the intervention and control arm, a sample size of 112 participants in each arm was required, assuming an expected mean value for sitting time of 500 ± 200 min per day. To obtain the desired statistical power, it was assumed that a sample size of 300 participants, with 120 in each arm and accounting for an expected drop-out rate of 20%, was appropriate. The sample size was calculated using STATA statistical software version 18 (StataCorp, College Station, TX, USA) [25,26,27,28]. We applied a non-probability sampling method. Here, we give a summary of the study methods. In brief, the study was performed in two places in the Marche region, Central Italy: one in the Diabetology Unit of the IRCCS INRCA Hospital in Ancona and the other in the Diabetology Unit of the ASUR Hospital of Fabriano. Before the start of the study, to reduce the variability among healthcare practitioners in terms of how they perform the intervention, a training event involving GPs, specialist physicians, and exercise specialists was performed. In this meeting, the patient’s inclusion/exclusion criteria, recruitment strategies, ERS, and WBA were explained. The first patient was enrolled on 9 December 2015. The last visit of the last patient was performed on 14 October 2018. The assessments were performed within the medical facilities. The enrolled patients were assigned to the intervention or control arm. The exercise training program was carried out within third-party fitness facilities. Older T2D patients who wanted to participate voluntarily in the study and met the inclusion criteria had to sign a written informed consent before being recruited to the trial. Trial staff had to withdraw enrolled patients if they no longer met the inclusion and exclusion criteria. The data were collected using standardized case report forms (CRFs). The assessments were carried out by healthcare professionals who followed the patients during the physical activity program at four time points: baseline coinciding with enrolment (T1), 6 months from baseline (T2), 12 months from baseline (T3), and 18 months from baseline (T4) (Figure 1). Complete information on the trial is reported in the CONSORT 2010 checklist and is available in Supplementary Materials. 

The study implemented the ERS for older T2D patients. The ERS aimed to promote PA and adoption by collaboration involving GPs, specialist physicians, exercise specialists, and patients. The ERS started with a GP referral of eligible T2D patients to the specialist physician of the Diabetology Unit. The specialist physician assessed the patient’s clinical status and referred them to an exercise specialist at a third-party facility. The exercise specialist designed a personalized exercise program. The ERS included a WBA designed for the study. The WBA allowed the specialist physician and GP to monitor each patient’s adherence to the exercise prescription. It served as a platform for recording, storing, processing, and using data during the trial. The WBA developed was accessible online for the patients and trial staff. It served as the platform for recording, storing, processing, and utilizing trial data. The WBA aimed to enhance collaboration among study stakeholders with varying roles and locations. Its web-based nature enabled stakeholders to access content via web browser, regardless of the device used. Enrolled patients could find, via WBA promotional information, an active lifestyle, physical activity, healthy diet guidelines, and self-management strategies for T2D older adults. Patients in the intervention arm could access personalized training data, including protocol adherence, average exercise intensity, duration, and motivational feedback. The WBA also provided clinical and non-clinical data to general practitioners and specialist physicians, facilitating patient monitoring and compliance feedback.

Monitoring was planned at baseline and at 6, 12, and 18 months from then; considering that the theory of change points out that a relapse phase is natural in the process of behavioral change, we wanted to verify if there were any differences after each consecutive 6 months of the program, each consisting of 3 months of supervised exercise followed by 3 months of unsupervised training.

### 2.2. Eligibility Criteria

#### 2.2.1. Inclusion Criteria

- Age range 65 to 74 years.
- Subjects with T2D according to ADA criteria [11].
- Physically inactive or insufficiently active by the International Physical Activity Questionnaire (IPAQ) short form [29].

#### 2.2.2. Exclusion Criteria

- Receiving any drug that could affect heart rate, such as β-blockers.
- Chronic obstructive pulmonary disease.
- Severe cardiovascular disease (including New York Heart Association class III or IV congestive heart failure), clinically significant valvular disease, history of cardiac arrest, presence of an implantable defibrillator, or uncontrolled angina.
- History of myocardial infarction, transient ischemic attack, or stroke in the six months before enrolment.
- Life expectancy <6 months.
- A condition that might harm the subjects’ health during the participation in the trial.

### 2.3. Intervention 

At enrolment, healthy lifestyles, informative material and behavioral counseling were provided to all subjects

#### 2.3.1. Intervention Arm

A structured physical activity program under the supervision of qualified personnel included three training sessions per week in a fitness center. The expertly supervised exercise program included moderate-intensity aerobic exercise (comparable to commonly used intensities in walking/jogging and cycling). Aerobic exercise intensity was prescribed and monitored according to heart rate (HR). For each patient, the heart rate reserve (HRR), which is the difference between resting heart rate (HRrest) and maximum heart rate (HRmax), was used to apply the percentage of target heart rate (%HRtarget) prescribed for a given exercise period. Patients’ HRrest was directly measured, while the HRmax was estimated using the equation proposed by Gellish et al. [30]. The exercise session’s target heart rate (HRtarget) for each patient was calculated in beats/minute as follows: HRtarget = [(HRmax − HRrest) × %HRtarget] + HRrest. It accurately reflects the percentage of reserve values of oxygen uptake ($\dot{V}$O2R) and allows for the correction of the exercise intensity for the HRrest.

The exercise intensity and duration differ for period 1 (months 1–3), period 3 (months 7–9), and period 5 (months 13–15) (Table 1). The exercise specialist gradually increases exercise duration and intensity each trimester [31].

During period 2 (months 4–6), period 4 (months 10–12), and period 6 (months 16–18), participants were encouraged to exercise independently (self-contained physical activity), receiving regular follow-up reminders from study personnel (Table 1). They were asked to write their exercise duration, HR average, and perceived exertion.

#### 2.3.2. Control Arm

Patients assigned to the control arm were only advised by the trained physician on the physical activity schedule and by nutrition/diets on the proper nutrition for people with T2D. The subjects received written, informative material in the form of brochures.

### 2.4. Outcome Measures

#### 2.4.1. Primary Outcome

The primary outcome of the TRIPL-A study is the difference between the intervention and the control arm after 6, 12, and 18 months from baseline in terms of changes in the values of ST (sitting time) and MET (metabolic equivalent) average weekly data obtained through the International Physical Activity Questionnaire short-form questionnaire IPAQ-SF [27,32].

#### 2.4.2. Secondary Outcomes

The secondary outcomes measured the difference between the intervention and control arm of the following parameters:

- Evaluation of physical performance by Long Distance Corridor Walk (LDCW) test [33]. The test has also been validated for estimating maximum oxygen consumption (VO_2max_) in the 60–91 age group. The test includes a first warm-up phase of 2 min and a second phase in which the subject is asked to walk as fast as possible, back and forth, in a 20 m long path, for 400 m.
- Fasting glucose, HbA1c.
- Body mass index (BMI = Kg/m^2^), waist and hip circumference, systolic and diastolic blood pressure (mmHg), resting heart rate (beats/min).
- Quality of life is measured using the Euro-QoL Index (EQ-5D-5L) [34].
- Sleep–wake disturbances are measured by the Pittsburgh Sleep Quality Index (PSQI) [35].

### 2.5. Statistical Analysis

For each study outcome at baseline, the intervention and control arm were compared using independent sample *t*-tests for continuous variables and chi-squared tests for categorical variables. Continuous variables were expressed as means with standard deviations (SDs), and discrete variables as numbers with percentages (%). A repeated measures ANOVA was used to compare each study outcome between the intervention and control arm at the four different time points, followed by post hoc pairwise comparisons when a significant interaction was found. For all tests, two-sided *p* values with an α level of significance of 0.05 were used. Bonferroni’s criterion was used to adjust the overall α level to correct for multiple tests. SPSS Statistics (IBM) software version 27 was used to perform data analyses.

## 3. Results

A total of 305 older adult subjects with T2D were recruited. Two hundred thirty-two subjects were recruited at the Diabetology Unit of the INRCA POR of Ancona and seventy-three at the Diabetology Unit of the Fabriano Hospital. The general and demographic information of the subjects at baseline is reported in Table 1. The mean age of patients was 68.7 years (range 65–74 years), of whom 53.8% were women. Participants were mostly married or cohabitating (77.2%), with mainly high school education (39%). Most had no difficulty reaching the TRIPL-A gym by their means (86.7%). No significant differences were found between the intervention and control group at baseline for the characteristics reported in Table 2.

Clinical information at baseline is described in Table 2. The baseline characteristics of the entire participant group were sedentary (MET mean 770.5), overweight (BMI mean 29.6 Kg/m^2^), with high waist circumference (mean 104.2 cm) and hips (mean 108.8 cm), with moderate metabolic control (mean HbA1c 7.2%), with hypercholesterolemia in 40.1% of the subjects, but with controlled cholesterol levels (average 170.1 mg/dL). The subjects were mainly hypertensive (55.6%) with controlled blood pressure values (mean systolic 135.2 mm/Hg; mean diastolic 74.7 mm/Hg). 15.7% of the participants were smokers. Significant differences between intervention and control groups were not found for most evaluated parameters. Differences between the two groups were found for hypertension (*p* = 0.031). 

During the study, no serious adverse events were registered. The total number of drop outs was 19% (31 subjects in the intervention and 27 in the control group). The motivation of 58 T2D patients drop out were: 30 failures to adhere to the intervention, 9 for personal reasons, and 19 for health problems. In particular, subjects that drop out for health reasons reported the following conditions: 4 back pain, 3 depressions, 2 ankle pain, 2 pain in the knees, 1 cholecystectomy, 1 myocardial infarction, 1 atrial fibrillation, 1 breast cancer, 1 saphenectomy, 1 hysterectomy, 1 colon adenocarcinoma, and 1 thyroidectomy.

The outcomes analyses were performed only in the subjects with >70% compliance in terms of performed training sessions with respect to expected ones during the supervised physical activity period (*n* = 92). In the non-supervised training periods, participants were trained independently and asked to enter the actual workout performed into the WBA at the end of each self-monitored exercise session. However, the adherence to the exercise program during the supervised periods was higher (mean 2.4 ± 0.7 days/week) than during the unsupervised periods (mean 2.1 ± 0.6 days/week). Considering that patients’ activities were supervised in the gym, the adherence data were verified instead of the adherence during the unsupervised period that was self-reported by the patients and, therefore, unverifiable. This doubt arises from the observation that in the intervention group, the ST decreased significantly from baseline only up to 6 months (*p* = 0.01), and the MET increased from baseline at six months (*p* = 0.01) only up to 12 months (*p* < 0.01), without further improvement of both parameters up to 18 months.

**Figure 2:**
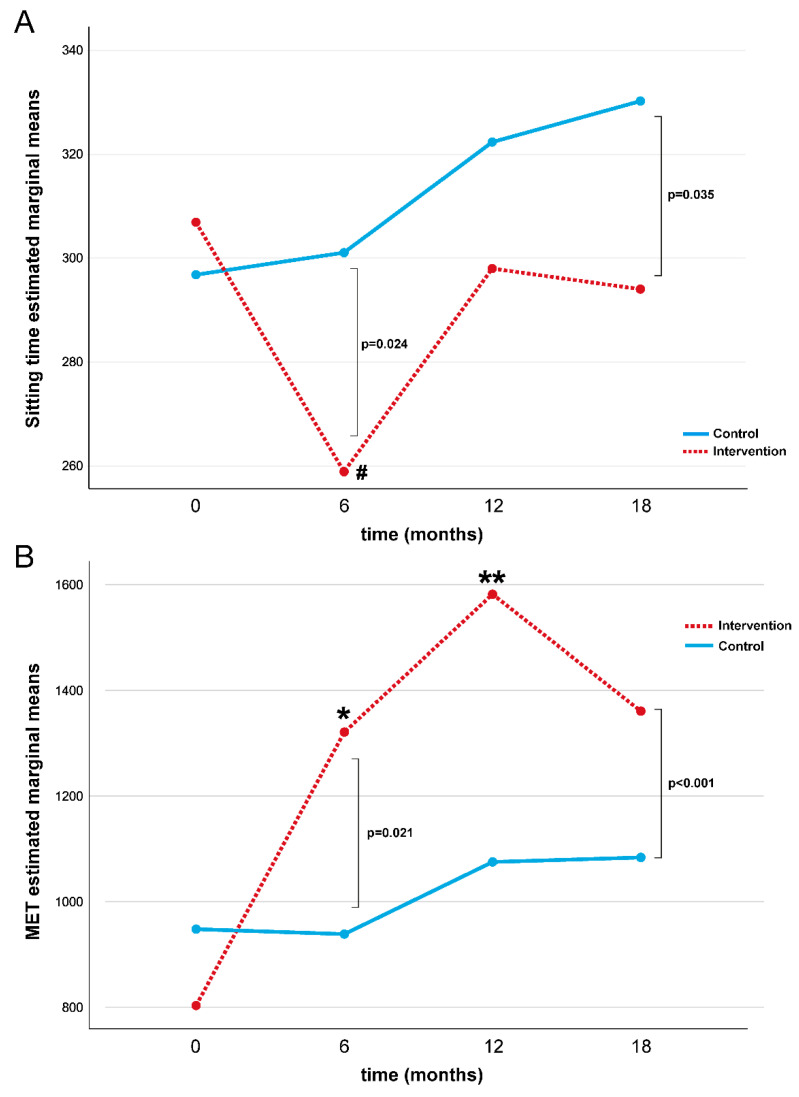
Repeated measures ANOVA test for ST (**A**) and MET (**B**) during the study for intervention and control groups. Note: MET = Metabolic Equivalent; ^#^ baseline vs. 6-month Bonferroni post hoc comparison in intervention group *p* = 0.001; * baseline vs. 6-month Bonferroni post hoc comparison in intervention group *p* = 0.01; ** baseline vs. 12-month Bonferroni post hoc comparison in intervention group *p* < 0.01.

A repeated-measures ANOVA determined that ST differed significantly across the four-time points compared to the placebo control group (*p* = 0.028, Table 3 and Figure 2A). In the intervention group, a post hoc pairwise comparison using the Bonferroni correction showed a decreased ST between the baseline and 6-month follow-up assessments (*p* = 0.001), while no significant differences were found at 12 and 18 months. No statistical differences in ST were found in the control group over time.

A repeated-measures ANOVA determined that the MET differed significantly across the four-time points compared to the placebo control group (*p* = 0.004, Table 3 and Figure 2B). In the intervention group, a post hoc pairwise comparison using the Bonferroni correction showed an increased MET value during the first twelve months of intervention (baseline vs. 6 months: *p* = 0.01 and baseline vs. 12 months: *p* < 0.01), while no difference in the MET value was found at18 months. No statistical differences were found in MET values in the control group over time. No significant differences were found for the intervention and control group for glucose, HbA1c, LDCW time, LDCW VO_2_, EQ-5D-5L test, and PSQI (Table 4).

Subjects excluded from the analysis due to missing data showed no statistical differences in clinical information and parameters assessed at baseline compared with the studied group, except for females who had a higher dropout rate of 24.6 percent compared with males of 13.4 (chi-squared = 6.33, *p* = 0.012). The results of repeated-measures ANOVA for ST and the MET were confirmed in sensitivity analyses performed only on females and in study subjects after excluding patients with retinopathy and hypertension (Table 5).

## 4. Discussion

T2D affects multiple organs and systems, such as the heart, brain, kidneys, and nerves, and presents a growing health problem [36]. Consequently, T2D significantly impacts the health system [8]. One way that problem could be met is by applying an education program to modify urbanization-related lifestyle habits. Professional health education, such as the self-management of T2D, in terms of diet and PA could help change unhealthy lifestyle habits [8]. For example, it has been seen that ERSs could be an efficient model for promoting PA in primary care settings [19].

The results of the TRIPL-A study have demonstrated that discontinuously supervised aerobic training can counteract sedentary behavior and increase the MET assessed by an IPAQ test in the older adult population with T2D. However, this intervention could improve lifestyle trends only within the first year of application. The minutes/week of ST by the seventh question of the IPAQ-SF test significantly decreases at 6 months from baseline. The MET value obtained by the IPAQ-SF test significantly increases up to 12 months of intervention. No additional improvements were observed after one year of intervention and even the described improvements were maintained for up to 18 months.

Longitudinal evaluations showed that the MET levels increased in the intervention group, and ST decreased more than in the controls. The study’s result showed that the exercise reference scheme, ERS, proposed by the TRIPL-A project led to an improvement in the habits of older adult diabetic subjects who switched to being more active. The discontinuously supervised aerobic training program was one of the study’s special features. An exercise specialist supervised the training sessions during the first, third, and fifth three-month periods. In the remaining periods, the subjects exercised at home on their own. This discontinuous exercise pattern involved periods of supervised exercise in the gym and independent exercise alone at home. The primary objective of the study was to educate diabetic patients to be less sedentary and to become accustomed to a more active lifestyle. The physical activity prescribed during the supervised training periods followed the guidelines for diabetic subjects. However, the physical activity was not monitored by accelerometers that, as previously described, could provide a more reliable assessment than a questionnaire [37]. This could represent a limitation of the study. Another way to make the intervention more robust could be using telemedicine when patients exercise alone at home. A recent qualitative meta-synthesis revealed that most patients who utilized remote devices felt motivated to manage their lifestyles. In fact, subjects experienced reassurance due to close monitoring and improved communication [38].

The interruption in the positive trend of participants’ improvement can be explained by the fact that, after an initial phase characterized by concrete steps to modify behavior, the second stage of maintenance may be challenged by reverting to old habits and can last for an extended period. Considering this, we may assume that participants experienced difficulties maintaining a long-term commitment. A recent systematic review demonstrated that various intervention approaches were effective in increasing PA only during the intervention period [39].

While continuing to attend the gym in the last phase of the program, carrying out the necessary actions may have become challenging for various reasons, thus reducing the impact of the intervention. In addition, even if we did not find significant improvement in QoL measured by EQ-5D-5L, physical exercise habit is known to improve long-term health status and positively affect QoL.

According to the theory of change, a relapse phase is natural. Relapse is not a phase in itself but is considered a common part of the change process [40]. It occurs when people return to their old behaviors after making progress. Relapse can be discouraging, but it is seen as a normal part of the change process, and people can use it as an opportunity to learn and adjust their strategies.

One of the main insights of this theory is that behavioral change is not a linear process [41]. People can move back and forth between stages, and progress is not always constant. The model also highlights the importance of tailoring intentions and support to an individual’s specific stage of change. When older adults experience a setback in their commitment to exercise, it is important to act and provide support to help them overcome this challenging period. Some actions such as social support, psychological support and motivation, and providing resources can be taken [42]. Regarding social support, it could be possible to involve friends or family members of the subjects in their exercise routine. Exercising with others can be more motivating and enjoyable [43]. Encouraging participation in exercise groups specifically designed for older adults could be useful [44]. In cases of severe setbacks or if older adults are facing emotional difficulties related to the setback, it may be appropriate to involve a mental health professional to provide psychological support [40,45,46]. Finally, it could be ensured that older adults have access to the necessary resources for exercise, such as suitable fitness facilities, proper equipment, and comfortable clothing. Our results suggest that it would be interesting for future RCT studies to involve a mental health professional figure in the team during the discontinuous period of intervention to help patients overcome a relapse phase and contribute a non-sedentary lifestyle to T2D patients with personalized support. The results of the TRIPL-A study derive from a robust clinical trial design. However, the limitation of this study is that only diabetic patients were considered. Further research should validate the results on an elderly population with other age-related chronic diseases.

## 5. Conclusions

In conclusion, the TRIPL-A study aimed to educate patients about a more active lifestyle. The intervention has been shown to reduce sitting time and increase physical activity in the first year of implementation. The theory of change provides that relapse is a common part of the change process. It occurs when people return to their old behaviors after making progress. Considering the benefits on individual and socio-economic levels that physical activity could bring, it may be helpful to involve a mental health professional figure in standard clinical practice to provide psychological support to older T2D patients to maintain healthy, non-sedentary lifestyles.

## Figures and Tables

**Table 1:** Supervised (S) and non-supervised (NS) aerobic training rate of progression for exercise session intensity and duration.

	Period 1	Period 2	Period 3	Period 4	Period 5	Period 6
	S	NS	S	NS	S	NS
Intensity (%HRR range)	40–50	50	45–55	55	50–60	60
Duration (minute range)	20–30	30	30–40	40	40–50	50

Notes: Values are indicative and were modified to the patient’s response to exercise; %HRR—heart rate reserve percentage.

**Table 2:** General, demographic, and clinical information of the participants at baseline.

Variable	Total (*n* = 305)	Control (*n* = 152)	Intervention (*n* = 153)	*p*
Gender female, *n* (%)	164 (53.8)	89 (58.6)	75 (49.0)	0.095
Age, mean ± SD	68.7 ± 4.1	68.5 ± 4.8	68.8 ± 3.3	0.650
Study degree, *n* (%)				0.707
Primary school diploma	88 (28.9)	42 (27.7)	46 (30.3)	
Middle school diploma	81 (26.4)	46 (30.1)	34 (22.4)	
High school diploma	119 (39.0)	55 (36.1)	64 (42.1)	
University degree	17 (5.7)	9 (6.0)	8 (5.3)	
Older adults can reach TRIPL-A gym independently by car, *n* (%)	264 (86.7)	127 (83.8)	137 (89.5)	0.147
Body mass index (kg/m^2^), mean ± SD	29.6 ± 6.0	29.6 ± 7.1	29.6 ± 4.7	0.975
Systolic blood pressure (mm/Hg), mean ± SD	135.2 ± 16.6	136.5 ± 18.2	133.9 ± 14.9	0.185
Diastolic blood pressure (mm/Hg), mean ± SD	74.7 ± 8.4	74.4 ± 8.9	75.0 ± 7.9	0.583
Heart rate (bpm), mean ± SD	76.0 ± 12.4	75.3 ± 11.7	76.6 ± 13.0	0.363
Waist circumference (cm), mean ± SD	104.2 ± 13.6	104.1 ± 14.0	104.2 ± 13.4	0.940
Hip circumference (cm), mean ± SD	108.8 ± 14.4	108.9 ± 15.4	108.7 ± 13.3	0.911
Arm circumference (cm), mean ± SD	30.4 ± 3.7	30.3 ± 3.7	30.6 ± 3.6	0.556
Calf circumference (cm), mean ± SD	37.1 ± 4.1	36.9 ± 4.2	37.4 ± 4.1	0.285
Fasting glucose, (mg/dL), mean ± SD	135.1 ± 41.3	136.2 ± 34.5	134.3 ± 36.7	0.832
Hba1c, (%), mean ± SD	7.2 ± 1.4	7.1 ± 1.5	7.3 ± 1.1	0.281
Total cholesterol, (mg/dL), mean ± SD	170.7 ± 32.5	165.5 ± 34.9	177.1 ± 28.4	0.059
Joint pain, *n* (%)	141 (46.2)	70 (46.1)	71 (46.4)	0.951
Pain in the lower limbs, *n* (%)	108 (35.4)	61 (40.1)	47 (30.7)	0.086
Age when T2D was diagnosed? mean ± SD	55.8 ± 14.0	55.7 ± 13.8	55.8 ± 14.2	0.933
Liver disease, *n* (%)	6 (2.0)	4 (2.7)	2 (1.3)	0.400
Hypercholesterolemia, *n* (%)	122 (40.1)	61 (40.0)	61 (40.1)	0.981
History of cancer, *n* (%)	10 (3.3)	4 (2.7)	6 (4.0)	0.534
Hypertension, *n* (%)	170 (55.6)	94 (62.0)	75 (49.3)	0.031
Osteoarthritis/Osteoporosis, *n* (%)	16 (5.3)	9 (6.0)	7 (4.6)	0.588
History of femur fracture, *n* (%)	4 (1.3)	2 (1.3)	2 (1.3)	0.989
Retinopathy, *n* (%)	39 (12.9)	25 (16.7)	14 (9.2)	0.053
Peripheral vascular disease, *n* (%)	13 (4.3)	10 (6.7)	3 (2.0)	0.082
Nephropathy, *n* (%)	12 (4.0)	9 (6.0)	3 (2.0)	0.073
Chronic kidney disease, *n* (%)	6 (2.0)	5 (3.3)	1 (0.7)	0.096
Smoke, *n* (%)				0.882
ex	134 (43.9)	67 (44.1)	67 (43.6)	
no	124 (40.5)	60 (39.3)	64 (41.6)	
yes	48 (15.7)	25 (16.6)	23 (14.8)	
EQ-5D-5L				
Movement ability problems, *n* (%)	51 (16.7)	31 (20.1)	20 (13.3)	0.115
Personal care problems, *n* (%)	13 (4.4)	8 (5.4)	5 (3.3)	0.381
Problems with usual activities, *n* (%)	30 (9.7)	17 (11.4)	13 (8.7)	0.430
Pain or discomfort, *n* (%)	136 (44.5)	70 (47.0)	66 (43.7)	0.569
Anxiety or depression, *n* (%)	109 (35.9)	58 (38.1)	52 (33.8)	0.440
State of health today, mean ± SD	73.2 ± 15.0	73.0 ± 15.1	73.5 ± 14.8	0.770
PSQI, mean ± SD	6.0 ± 3.4	6.2 ± 3.7	5.8 ± 2.9	0.315
LDCW time, mean ± SD	325.1 ± 64.0	331.4 ± 65.2	315.7 ± 61.4	0.071
LDCW VO_2max_, mean ± SD	17.1 ± 3.8	16.8 ± 3.9	17.2 ± 3.3	0.079

Note: EQ-5D-5L = Euro-QoL-5D-5L test; PSQI = Pittsburgh Sleep Quality Index; LDCW time = Long Distance Corridor Walk; LDCW VO_2max_ = Long Distance Corridor Walk oxygen consumption.

**Table 3:** Baseline, 6, 12, and 18 months’ levels in ST and MET values by group allocation (intervention *n* = 92, control *n* = 125).

	Baseline	6th Months	12th Months	18th Months	F	*p* Group	F	*p* Time	*F*	*p* Interaction
ST (min)					2.063	0.152	7.831	<0.001	3.099	0.028
Intervention	306.8 (164.6)	251.0 (107.5)	273.6 (108.5)	283.1 (103.0)						
Control	296.7 (180.5)	301.5 (158.5)	323.0 (171.2)	331.3 (143.3)						
MET (min/week)					8.218	0.005	16.471	<0.001	4.670	0.004
Intervention	803.3 (967.2)	1321.2 (1183.9)	1581.6 (1404.2)	1360.9 (1117.1)						
Control	948.1 (1124.2)	938.6 (1065.2)	1075.3 (1149.5)	1083.8 (1296.3)						

Note: ST = Sitting time; MET = Metabolic Equivalent.

**Table 4:** Baseline, 6, 12, and 18 month levels of secondary outcomes parameters by group allocation (intervention *n* = 92, control *n* = 125).

	Baseline	6th Month	12th Month	18th Month	F	*p* Group	F	*p* Time	F	*p* Interaction
Glucose (mg/dL)					3.351	0.068	4.962	0.002	1.251	0.292
Intervention	140.7 (30.5)	144.4 (36.9)	134.2 (36.3)	133.1 (28.4)						
Control	134.9 (27.4)	130.6 (26.8)	133.3 (27.2)	129.2 (24.7)						
HbA1c (%)					0.905	0.342	5.063	0.002	0.998	0.394
Intervention	7.3 (1.1)	7.0 (1.0)	7.0 (1.0)	7.1 (1.0)						
Control	7.1 (1.0)	7.0 (1.0)	7.0 (1.0)	7.0 (0.9)						
LDCW time					1.137	0.290	0.988	0.403	0.876	0.458
Intervention	318.7 ± 60.7	304.9 ± 43.0	306.8 ± 46.3	301.1 ± 42.5						
Control	332.6 ± 67.1	321.3 ± 48.0	325.5 ± 41.8	326.7 ± 50.4						
LDCW VO_2max_					1.311	0.311	2.110	0.115	0.175	0.913
Intervention	17.5 ± 3.6	18.2 ± 2.4	18.0 ± 2.7	18.4 ± 2.4						
Control	16.6 ± 4.0	17.3 ± 2.8	17.0 ± 2.5	16.5 ± 2.1						
PSQI					0.997	0.997	0.733	0.534	0.406	0.749
Intervention	5.8 ± 2.9	6.0 ± 3.2	6.2 ± 3.4	6.5 ± 3.1						
Control	6.2 ± 3.7	5.7 ± 3.4	6.1 ± 3.5	6.0 ± 3.2						
EQ-5D-5L					1.282	0.280	1.303	0.274	0.696	0.555
Intervention	73.5 ± 14.6	74.0 ± 15.0	71.5 ± 17.2	75.5 ± 13.5						
Control	73.0 ± 15.1	72.4 ± 16.0	71.1 ± 16.7	71.0 ± 14.7						

Note: HbA1c = Glycosylated hemoglobin; LDCW time = Long Distance Corridor Walk; LDCW VO_2max_ = Long Distance Corridor Walk oxygen consumption; PSQI = Pittsburgh Sleep Quality Index; EQ-5D-5L = Euro-QoL-5D-5L test.

**Table 5:** Sensitivity analysis exploring the changes over the time of ST and MET in women, excluding subjects with diabetic complications and hypertension.

	Women (*n* = 164)		Excluding Subjects with Diabetic Complications (*n* = 167)		Excluding Subjects with Hypertension (*n* = 96)	
	F	*p* Value	F	*p* Value	F	*p* Value
ST (min)						
Group	0.404	0.526	0.238	0.626	0.211	0.647
Time	5.267	0.002	6.681	<0.001	4.141	0.007
Interaction	2.688	0.049	3.326	0.020	2.677	0.048
MET (min/week)						
Group	7.205	0.008	3.369	0.020	2.300	0.133
Time	17.818	<0.001	13.824	<0.001	6.716	<0.001
Interaction	2.839	0.041	6.100	0.014	2.825	0.039

Note: ST = sitting time; MET = metabolic equivalent.

## Data Availability

Data are available upon reasonable request to the corresponding author.

## Supplementary Materials

The following supporting information can be downloaded at: https://www.mdpi.com/article/10.3390/healthcare12080848/s1, CONSORT 2010 checklist of information to include when reporting a randomised trial.
